# Increased Adaptive Variation Despite Reduced Overall Genetic Diversity in a Rapidly Adapting Invader

**DOI:** 10.3389/fgene.2019.01221

**Published:** 2019-11-26

**Authors:** Daniel Selechnik, Mark F. Richardson, Richard Shine, Jayna L. DeVore, Simon Ducatez, Lee A. Rollins

**Affiliations:** ^1^School of Life and Environmental Sciences (SOLES), University of Sydney, Sydney, NSW, Australia; ^2^Evolution and Ecology Research Centre, School of Biological, Earth, and Environmental Sciences, University of New South Wales, Sydney, NSW, Australia; ^3^Deakin Genomics Centre, School of Life and Environmental Sciences, Deakin University, Geelong, VIC, Australia; ^4^Centre for Integrative Ecology, School of Life and Environmental Sciences, Deakin University, Geelong, VIC, Australia

**Keywords:** *Bufo marinus*, Rhinella marina, evolution, invasive species, ribonucleic acid sequencing, genetic paradox of invasion

## Abstract

Invasive species often evolve rapidly following introduction despite genetic bottlenecks that may result from small numbers of founders; however, some invasions may not fit this “genetic paradox”. The invasive cane toad (*Rhinella marina*) displays high phenotypic variation across its introduced Australian range. Here, we used three genome-wide datasets to characterize their population structure and genetic diversity. We found that toads form three genetic clusters: 1) native range toads, 2) toads from the source population in Hawaii and long-established areas near introduction sites in Australia, and 3) toads from more recently established northern Australian sites. Although we find an overall reduction in genetic diversity following introduction, we do not see this reduction in loci putatively under selection, suggesting that genetic diversity may have been maintained at ecologically relevant traits, or that mutation rates were high enough to maintain adaptive potential. Nonetheless, toads encounter novel environmental challenges in Australia, and the transition between genetic clusters occurs at a point along the invasion transect where temperature rises and rainfall decreases. We identify environmentally associated loci known to be involved in resistance to heat and dehydration. This study highlights that natural selection occurs rapidly and plays a vital role in shaping the structure of invasive populations.

## Background

The genetic paradox of invasion (Allendorf, 2003) describes a phenomenon that challenges widespread evidence of the relationship between genetic diversity and adaptive potential. High genetic diversity within a population is beneficial because it likely underlies phenotypic variation, allowing the population to respond to selection imposed by environmental change (Reed and Frankham, 2003; Frankham, 2005). Furthermore, a greater number of alleles confers an increased frequency of heterozygosity, which is often associated with population fitness (Reed and Frankham, 2003). Small or isolated populations with low genetic diversity have been shown to suffer declines due to inbreeding depression and the associated reduction of individual fitness (Westemeier et al., 1998; Madsen et al., 1999; Blomqvist et al., 2010). Conservation efforts to salvage such populations by introducing individuals from allopatric populations (thereby introducing new alleles; “genetic rescue”) have been successful, suggesting that the maintenance of genetic diversity can be crucial for population viability (Westemeier et al., 1998; Madsen et al., 1999).

Despite the fact that invasive populations are thought to undergo genetic bottlenecks due to the translocation of a small number of founders from their native range to an introduced range (Barrett and Kohn, 1991; Allendorf, 2003), invasive species are also characterized by their ability to establish and spread in their introduced ranges. This could be facilitated by factors such as empty ecological niches or a lack of natural enemies (Colautti et al., 2004). However, invasion success is also commonly linked to rapid evolution, including adaptation to novel environmental conditions over short timescales (Franks et al., 2012; Colautti and Barrett, 2013; Sultan et al., 2013). Additionally, some invaders exhibit novel phenotypic traits that enhance invasive potential, such as increased growth and dispersal rates (Whitney and Gabler, 2008; White et al., 2013). There are many examples of evolutionary change during invasion without high levels of genetic diversity (Rollins et al., 2013).

Although low genetic diversity may limit the ability of an invasive population to respond to natural selection, rapid evolution can also occur through non-adaptive processes. 1) Genetic drift may occur on range edges, reducing genetic diversity across an introduced range (Rollins et al., 2009); 2) spatial sorting is the separation of individuals within a range-expanding population along phases of their expansion based on their dispersive capabilities, and was first characterized in Australian cane toads (Shine et al., 2011). Due to spatial sorting, the invasion front may be inhabited exclusively by the individuals with the highest dispersal rates (even if they are less fit than individuals with low dispersal rates) because they have arrived first and can only breed with each other (Shine et al., 2011), resulting in a geographic separation of phenotypes (Shine et al., 2011; Hudson et al., 2016). 3) Admixture or hybridization may occur between individuals from different introductions or sources (Mader et al., 2016).

It has recently been suggested that the genetic paradox of invasion may be rare; some invasive populations do not suffer a reduction in genetic diversity during introduction, and others do not face novel adaptive challenges in their introduced ranges (Estoup et al., 2016). Furthermore, some invasive systems demonstrate a “spurious” paradox due to inadequate estimation of genetic diversity (i.e., too few markers), or to maintenance of genetic diversity only at ecologically relevant traits despite an overall reduction, or to a reduction in genetic diversity resulting from natural selection rather than from genetic bottlenecks (Estoup et al., 2016). These ideas can be tested with genome-wide data because many markers are sampled, and because bioinformatics pipelines allow for selection tests and the identification of loci that may underlie ecologically relevant traits (Wellband et al., 2018; Willoughby et al., 2018).

**Figure 1:**
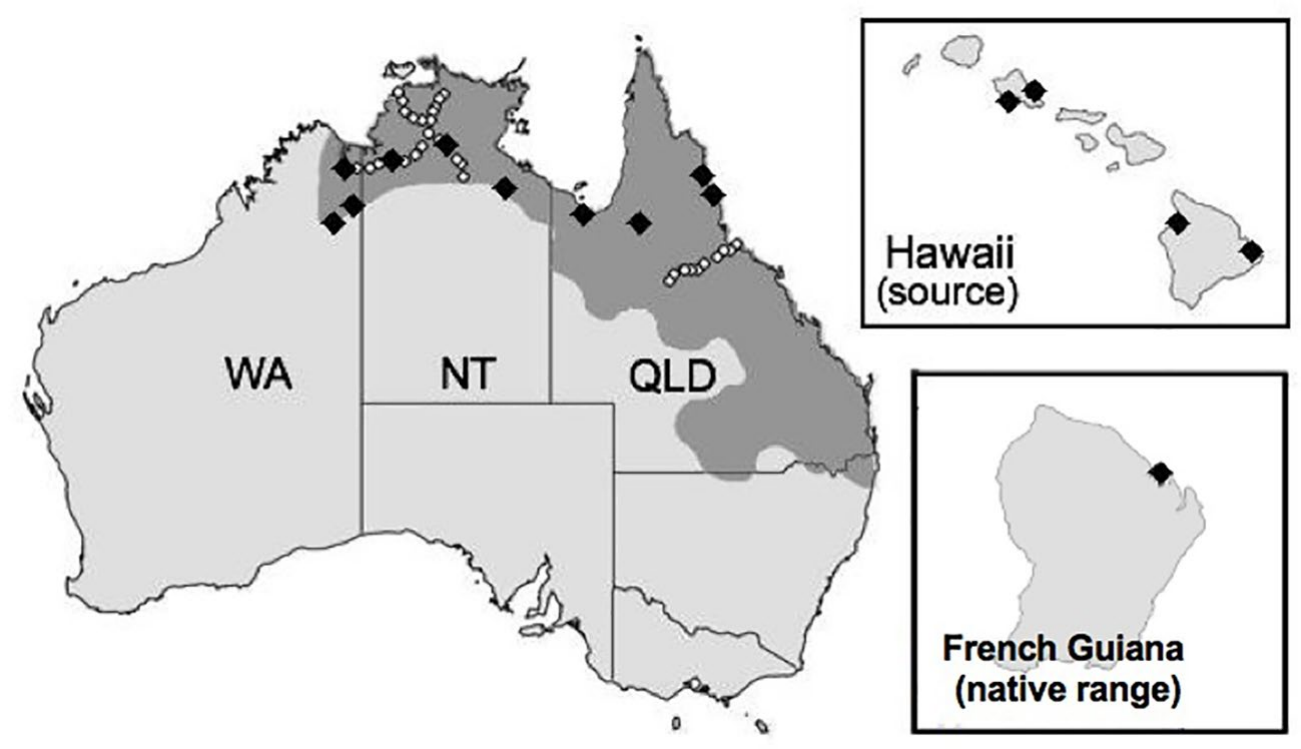
Map of two invaded territories (Australia and Hawaii) and the native range (French Guiana) of the cane toad (*Rhinella marina*). The shaded region represents the toad's current Australian range, which is continuing to expand. Black diamonds in French Guiana, Hawaii, and Australia indicate collection sites for our RNA sequence experiment. White circles in Australia indicate collection sites for the restriction site-associated DNA sequencing experiment by Trumbo et al. (2016).

The Australian cane toad (*Rhinella marina*) is an invader that has exhibited rapid evolution despite apparently low genetic diversity (Rollins et al., 2015). Cane toads were serially translocated from French Guiana and Guyana to Martinique and Barbados, to Jamaica, to Puerto Rico, to Hawaii before 101 founders were introduced from the Hawaiian island of Oahu to Queensland (QLD), Australia in 1935 (Turvey, 2009). Toads have since spread westward through the Northern Territory (NT) into Western Australia (WA; Figure 1).

Prior to reaching Australia, toads were exposed to tropical environments with relatively high mean annual rainfall (3,200 mm in French Guiana, 1,200–2,000 mm in Hawaii) and high temperatures (26°C in French Guiana, 22–24°C in Hawaii) (Hijmans, 2015). However, the Australian range is heterogeneous in several environmental factors; on average, sites in QLD are more similar to the native range with respect to aridity, receiving more annual rainfall than sites in the NT and WA (2,000–3,000 mm in QLD, 400–1,000 mm in NT and WA), and have lower annual mean temperatures (21–24°C in QLD, 24–27°C in NT and WA; **Figure S1**) (Bureau of Meteorology, A.G, 2018). Heritable phenotypic differences in behavior (Gruber et al., 2017), thermal performance (Kosmala et al., 2018a), morphology (Hudson et al., 2016; Hudson et al., 2018), and immune function (Brown et al., 2015c) have been documented in cane toads at different localities across the Australian range. Nonetheless, surveys have reported low levels of genetic diversity based on microsatellites (Leblois et al., 2000) and MHC (Lillie et al., 2014). Furthermore, there was only one mitochondrial haplotype in the *ND3* gene of 31 individuals sequenced from Hawaii and Australia (Slade and Moritz, 1998).

Here, we assessed genetic diversity and population structure using single nucleotide polymorphisms (SNPs) identified in RNA sequencing (RNA-Seq) data from samples originating from French Guiana (the “native range”), Hawaii (the “source”), QLD (the “range core”), NT (“intermediate” areas), and WA (the “invasion front”) to test whether the genetic paradox is evident in the cane toad invasion, and to investigate the evolutionary dynamics during introduction. We predicted high divergence between native and invasive populations due to a combination of genetic drift, selection, and spatial sorting, but little genetic differentiation within invasion phases due to a putative lack of standing genetic diversity. Because of the increase in aridity at intermediate areas and the invasion front, we predicted that loci involved in thermal tolerance would be under selection, which may underlie the success that toads have had dispersing through these areas.

RNA-Seq data provide genome-wide information, but are limited to transcribed regions of the genome (Davey and Blaxter, 2011). RNA-Seq data may therefore contain variants that exhibit strong signals for both demography and selection. Conversely, reduced representation sequencing approaches such as restriction site-associated DNA sequencing (RADSeq) provide genome-wide information on a subset of all coding and non-coding sequences (only SNPs near restriction enzyme cleavage sites are detected); because non-coding sequences make up most of the genome, SNPs detected using this technique are more likely to represent neutral variation (Wang et al., 2009). To focus on how selection may shape genomic diversity in invasive toads, we compared the results from our RNA-Seq data to those from a publicly available RADSeq dataset (Trumbo et al., 2016) that we reanalyzed.

## Materials and Methods

### Sample Collection, Ribonucleic Acid Extraction, and Sequencing

We collected samples from French Guiana, the Hawaiian Islands, and across Australia (**Figure 1**; **Table S1**). All procedures involving live animals were approved by the University of Sydney Animal Care and Ethics Committee (2014/562) and the Deakin University Animal Ethics Committee (AEX04-2014). We excised whole spleen tissue (sample sizes in **Table S1A**) and whole brain tissue (sample sizes in **Table S1B**) from female toads immediately after euthanasia, preserved samples in RNAlater (QIAGEN, USA), and stored them at −80°C.

We extracted RNA using the RNeasy Lipid Tissue Mini Kit (QIAGEN, USA) following the manufacturer's instructions, in conjunction with a genomic DNA removal step (QIAGEN, USA). We quantified total RNA using a Qubit RNA HS Assay (Life Technologies, USA). mRNA libraries were constructed using the TruSeq mRNA v2 sample kit (Illumina Inc., USA), including a 300 bp selection step. In total, we sequenced 46 spleens and 72 brains (**Tables 1** and **2**) across five lanes of Illumina HiSeq 2500. Capture of mRNA was performed using the oligo dT method, and size selection parameter choices were made according to the HiSeq2500 manufacturer's protocol. Overall, this generated 599 million (spleen) and 872 million (brain) paired-end 2 x 125-bp reads. Raw sequence reads are available in the National Center for Biotechnology Information (NCBI) short read archive (SRA) under the BioProject Accession PRJNA510261 (spleen data from French Guiana and Hawaii), PRJNA395127 (spleen data from Australia), and PRJNA479937 (all brain data).

**Table 1:** Expected heterozygosity (He), allelic richness (AR), and Shannon's Information Index (SI) estimated using single nucleotide polymorphisms from spleen RNA sequencing data on native and invasive populations of cane toads (*Rhinella marina*) at regions across French Guiana, Hawaii, and Australia.

Region	All loci (He, AR, SI)	Outlier F_ST_ loci (He, AR, SI)	Environmentally associated loci (He, AR, SI)
French Guiana (native, N = 8)	0.27, 1.74, 0.40	0.03, 1.25, 0.06	0.17, 1.53, 0.25
Hawaii (source, N = 10)	0.25, 1.72, 0.37	0.08, 1.26, 0.13	0.34, 1.90, 0.51
QLD (core, N = 10)	0.25, 1.73, 0.38	0.08, 1.29, 0.13	0.34, 1.91, 0.50
NT (intermediate, N = 8)	0.23, 1.69, 0.35	0.02, 1.07, 0.03	0.23, 1.73, 0.36
WA (front, N = 10)	0.23, 1.68, 0.34	0.02, 1.11, 0.04	0.21, 1.68, 0.33

QLD, Queensland; NT, Northern Territory; WA, Western Australia.

**Table 2:** Expected heterozygosity (He), allelic richness (AR), and Shannon's Information Index (SI) estimated using single nucleotide polymorphisms from brain RNA sequencing data on native and invasive populations of cane toads (*Rhinella marina*) at regions across French Guiana, Hawaii, and Australia.

Region	All loci (He, AR, SI)	Outlier F_ST_ loci (He, AR, SI)	Environmentally associated loci (He, AR, SI)
Hawaii (source, N = 18)	0.33, 1.97, 0.49	0.35, 1.87, 0.51	0.41, 2.00, 0.60
QLD (core, N = 18)	0.33, 1.98, 0.50	0.39, 2.00, 0.56	0.24, 1.92, 0.38
NT (intermediate, N = 18)	0.31, 1.95, 0.47	0.17, 1.66, 0.28	0.09, 1.52, 0.16
WA (front, N = 18)	0.29, 1.93, 0.45	0.12, 1.49, 0.19	0.07, 1.46, 0.12

QLD, Queensland; NT, Northern Territory; WA, Western Australia.

### Data Pre-Processing and Alignment

First, we conducted quality control (checking Phred scores, guanine-cytosine content, and adapter sequences) using FastQC v0.11.5 (Andrews, 2010b). We then processed raw reads using Trimmomatic v0.35 (Bolger et al., 2014) as follows: ILLUMINACLIP: TruSeq3-PE.fa:2:30:10:4 SLIDINGWINDOW:5:30 AVGQUAL:30 MINLEN:36 (**Tables S2A, B**).

As a reference, we used the annotated multi-tissue *R. marina* transcriptome (Richardson et al., 2018). We conducted per sample alignments of reads (FASTQ files) to the reference using STAR v2.5.0a (Dobin et al., 2013) in basic two-pass mode with default parameters, a runRNGseed of 777, and specifying binary alignment map (BAM) alignment outputs. Unmapped reads were discarded (**Tables S2A, B**). As STAR-generated BAM files lack read groups, we added them to our BAM files using the AddOrReplaceReadGroups tool in Picard Tools (Institute, 2018). To avoid making incorrect variant calls, we removed duplicate reads using the MarkDuplicates tool in Picard Tools. To split reads containing an N into individual exon segments, we used the SplitNCigarReads tool in the Genome Analysis Toolkit (GATK) v3.8.0 (Mckenna et al., 2010).

### Variant Calling and Filtering

To call SNPs and insertion-deletions (indels), we used the HaplotypeCaller tool in GATK (Mckenna et al., 2010) on our alignment (BAM) files. Variants required a minimum Phred-scaled confidence of 20 to be called (marked as passing the quality filters) and emitted (reported in the output), using the stand_call_conf and stand_emit_conf options.

To improve accuracy of SNP-calling, we performed this in Genomic Variant Call Format (“GVCF”) mode. By using this mode, we avoided missing SNPs at loci that match the reference in some but not all individuals. We then used the GenotypeGVCFs tool to merge the GVCF files, re-calculate genotype likelihoods at each SNP locus across all individuals, and re-genotype and re-annotate all SNP loci. The results were written to one merged VCF file. Although we initially genotyped all spleens and brains together, we discovered during downstream analyses that there was an effect of tissue type on population assignment; even from the same individuals, and using only SNPs from transcripts expressed in both tissues, spleen, and brain samples were assigned to separate populations. Thus, we genotyped spleens and brains separately, resulting in two merged VCF files, and subsequently kept these separate for all downstream analyses. We retained both datasets because each provides a unique benefit: the spleen dataset includes native range samples, but the brain dataset has more extensive sampling of the invasive populations.

We used the VariantFiltration tool to identify and filter “clusters” (sets of 3 SNPs that appear within a window of 35 bases) in each of our merged VCF files to avoid SNPs in linkage disequilibrium and also to filter variants with QualByDepth (QD) less than 2.0, depth of coverage (DP) less than 20.0, and allele frequency less than 0.05. This resulted in 803,489 SNPs from spleen data and 818,536 SNPs from brain data. We used bcftools (Li et al., 2009) to include only biallelic SNPs. We examined the results of filtering for minimum minor allele frequency (min MAF) thresholds of 0.01 or 0.05, and several missing data tolerance (MDT) thresholds (the maximum percentage of individuals in the dataset in which a genotype for a locus can be absent without that locus being filtered out). Our population structure results were consistent across min MAF and MDT thresholds, so we ultimately chose to filter our data at min MAF = 0.05 and MDT = 0% (no missing data tolerated) because some downstream analyses cannot handle missing data. These filtering steps reduced the number of SNPs to 65,195 in spleen data and 35,842 in brain data (**Tables S1A, B**).

### Inference of Population Structure

We used PLINK (Purcell et al., 2007) to convert our VCF files to the Browser Extensible Data (BED) format. With this, we used fastStructure (Raj et al., 2014) to infer population structure using a variational Bayesian framework for calculating posterior distributions, and to identify the number of genetic clusters in our dataset (K) using heuristic scores (Raj et al., 2014). We ran the structure.py with 10 replicates for each K (K = 1 to 10). We then took the resulting meanQ files from fastStructure and plotted them using the pophelper package (Francis, 2016) in R (Team, 2016).

We performed a redundancy analysis (RDA) using the vegan package (Oksanen et al., 2018) in R. RDA visualizes both population structure and the effects that environmental variables may have in shaping it. To do this, we downloaded climatic data for all sites from the Bioclim database (Hijmans et al., 2005). Because these areas vary in aridity, we downloaded data on rainfall and temperature (averages from 1970 to 2000 of annual mean temperature, maximum temperature of the warmest month, minimum temperature of the coldest month, annual precipitation, precipitation of the wettest quarter, and precipitation of the driest quarter). We then used the vcfR package (Knaus and Grünwald, 2017) to convert our VCF files to the GENLIGHT format (readable by the vegan package).

### Identification of Candidate Loci Under Selection

Loci that are under natural selection may have abnormally high or low F_ST_ values, causing them to be outliers that can be detected by Bayescan v2.1 (Foll and Gaggiotti, 2008) using the multinomial-Dirichlet model. We searched for outliers between our three genetic clusters in spleen data (native range toads *versus* source/core toads *versus* intermediate/frontal toads) and our two genetic clusters in brain data (source and core toads *versus* intermediate and frontal toads).

Loci under natural selection also may have allele frequencies that are associated with environmental variables. Using the same climatic data on rainfall during the driest quarter and maximum temperature in the warmest month mentioned above, we performed a latent factor mixed model (LFMM) in the lfmm v2.0 package (Frichot and Francois, 2015) in R to test these associations (Benjamini-Hochberg-corrected p-values).

Because these scans reveal thousands of loci putatively under selection, and because outlier tests have high false positive rates (Whitlock and Lotterhos, 2015), we took a more conservative approach by cross-matching our list of F_ST_ outliers with each of our lists of environmentally associated loci. We further investigated only those loci with both an outlier F_ST_ value and an association with a putatively significant environmental variable (through LFMM).

### Evaluation of Genetic Differentiation and Diversity

To quantify levels of genetic differentiation and diversity, we computed basic statistics (Lischer and Excoffier, 2012) in the hierfstat (Goudet, 2005) and diveRsity (Keenan et al., 2013) packages in R (Team, 2016), including global F_ST_ and pairwise F_ST_ (by genetic cluster, invasion phase, and collection site), expected heterozygosity (He), and rarefied allelic richness (AR). We also computed Shannon's Information Index (SI) with the dartR package (Gruber et al., 2018). After calculating these measures of diversity across all loci (N = 65,195 from spleen data, N = 35,842 from brain data), we calculated the same measures in loci putatively under selection: those with outlier F_ST_ values (N = 648 from spleen data, N = 203 from brain data) and those associated with environmental variables (N = 4,179 from spleen data, N = 530 from brain data). In spleen data, this allowed us to investigate the hypothesis that genetic diversity is maintained at ecologically relevant traits even if genome-wide diversity is lost. In brain data, this allowed us to examine the effect of natural selection on genetic diversity within Hawaii and Australia. We used Kruskal-Wallis tests to assess the significance of the differences in genetic diversity.

### Annotation of Single Nucleotide Polymorphisms

To visualize the types of genomic regions in which our SNPs lie, we used SnpEff (Cingolani et al., 2012). We calculated the relative proportions of each type of SNP for both tissue types using the full dataset of SNPs, and our two selection candidate datasets described above. We then used the stats v3.5.0 R package to perform z-tests on the differences in proportions of SNP types between the full and selection candidate datasets. We expected differences in the proportions of certain types of SNPs; although a lower proportion of synonymous variants in the selection candidates may be expected because synonymous mutations do not result in a codon change, recent studies suggest that even these mutations may contribute to adaptive variation (Plotkin and Kudla, 2011; Bailey et al., 2014; Brule and Grayhack, 2017; Kristofich et al., 2018).

### Isolation by Distance

To examine the effects of geographic distance on genetic distance across the Australian range, we performed a Mantel test using ade4 v1.7-5 (Thioulouse and Dray, 2007) using brain data. Because we were focused on isolation by distance through range expansion after introduction (to Australia), samples from Hawaii and French Guiana were excluded. We used the dist function in R (Team, 2016) to calculate pairwise Euclidean distances and took their natural log. For the genetic distance matrix, we used collection site-based pairwise F_ST_, and then linearized these values using the formula: linearized F_ST_ = F_ST_/(1 – F_ST_). We performed four Mantel tests, using: 1) all loci, 2) loci with outlier F_ST_ values, 3) loci with outlier F_ST_ values and with an environmental association (temperature or rainfall), 4) loci with outlier F_ST_ values but without an environmental association. A significant result across all loci may suggest that the prominent driver of genetic structure is genetic drift. However, clinal variation in allele frequencies may also result from spatial sorting (particularly in loci underlying dispersal ability), or from selection associated with environmental clines; distinguishing these evolutionary processes is difficult, and may not be possible with a Mantel test. However, we performed multiple Mantel tests on different subsets of loci to attempt to separate the effects of genetic drift and spatial sorting.

### Acquisition, Processing, and Analysis of Restriction Site-Associated Deoxyribonucleic Acid Sequencing Dataset

Unlike RNA-Seq data, which consists of coding sequences only (Wang et al., 2009), RADSeq data include SNPs from across the entire genome, but only around sites that are cleaved by a selected set of restriction enzymes (Davey and Blaxter, 2011). We contrasted these two approaches using a publicly available RADSeq dataset (Trumbo et al., 2016), thus comparing loci potentially under selection (RNA-Seq) with those primarily constituting neutral variation (RADSeq). Individuals in the RADSeq dataset were collected from QLD (range core toward intermediate areas, N = 179) and the border of NT/WA (intermediate areas toward the invasion front, N = 441; **Figure 1**). We downloaded raw 100-bp reads from NCBI SRA under the BioProject Accession PRJNA328156.

We used Stacks v2.0 (Catchen et al., 2013) for all RADSeq data processing, including quality control, construction of a *de novo* assembly, alignment of matching DNA regions across samples, and SNP calling using a maximum likelihood framework. We allowed a maximum of five base mismatches between stacks within an individual and three base mismatches between stacks between individuals, resulting in 499,623 SNPs. We retained one random SNP per locus and investigated four combinations of parameter choices (min MAF = 0.05, MDT = 0.5; min MAF = 0.05, MDT = 0.2; min MAF = 0.01, MDT = 0.5; min MAF = 0.01, MDT = 0.2), ultimately choosing min MAF = 0.05 and MDT = 0.2. These filtering choices produced results that were most consistent with those reported by Trumbo et al. (2016) and with those of our RNA-Seq dataset. Filtering reduced the number of SNPs to 8,296.

We ran fastStructure using the same methodology as above. We also performed a discriminant analysis of principal components (DAPC) using adegenet (Jombart and Ahmed, 2011). We computed the same basic statistics for the RADSeq dataset as we did for our RNA-Seq datasets above. Because collection site coordinate metadata were unavailable for the RADSeq dataset, we did not perform a Mantel test using these data.

## Results

### Population Structure Across the Hawaiian-Australian Invasion

**Figure 2:**
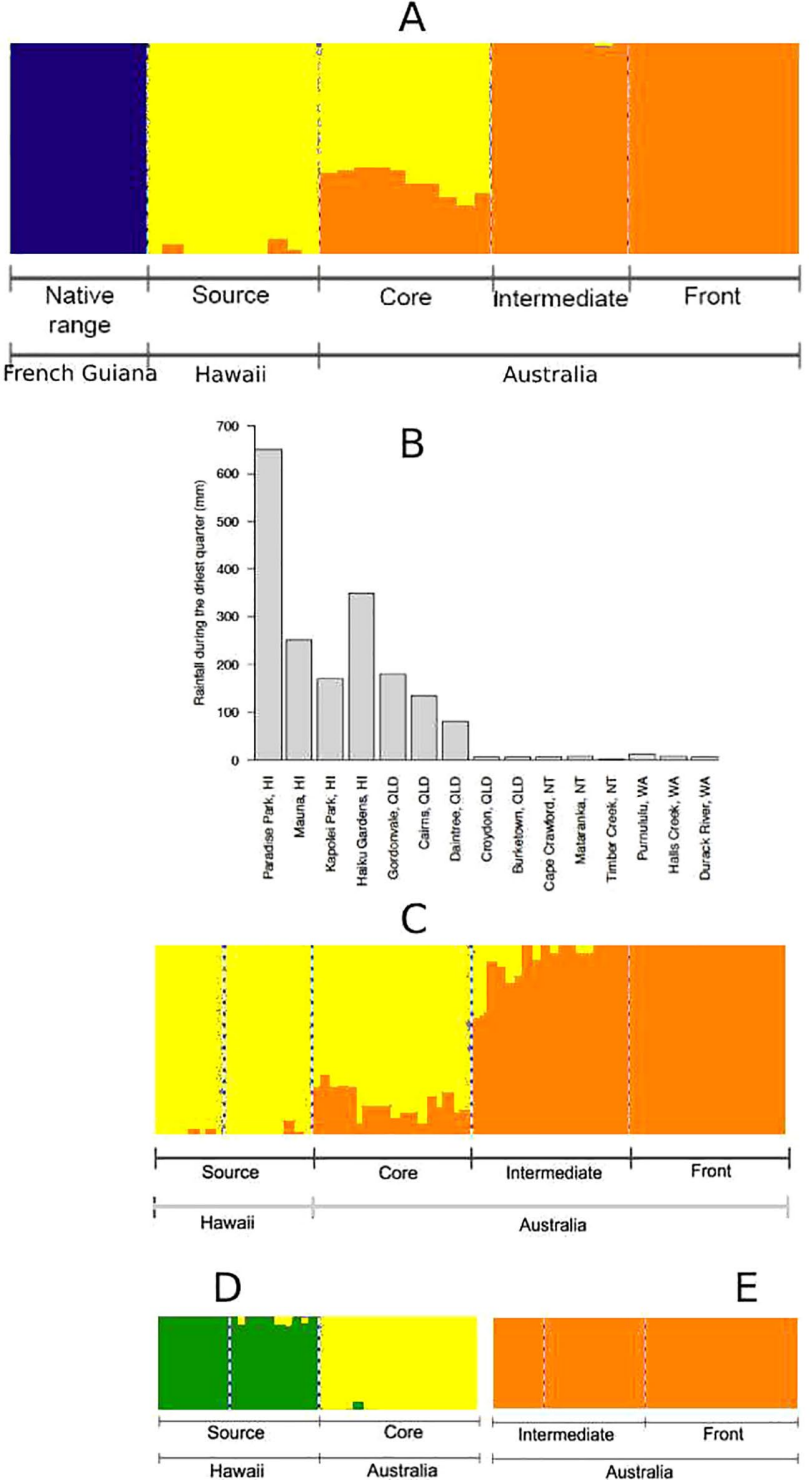
**(A)** Population structure of cane toads from French Guiana, Hawaii, and Australia, as inferred by single nucleotide polymorphisms (SNPs) from spleen RNA sequencing (RNA-Seq) data. **(B)** Rainfall during the driest quarter in each location across collection sites for invasive cane toads in French Guiana, Hawaii, and Australia. A similar environmental barrier occurs in temperature (**Figure S1**). **(C)** Population structure of invasive cane toads from Hawaii and Australia, as inferred by SNPs from brain RNA-Seq data. **(D)** Substructure within toads from Hawaii (HI) and Queensland (QLD) as inferred by SNPs from brain RNA-Seq data. **(E)** Substructure within toads from Northern Territory (NT) and Western Australia (WA) as inferred by SNPs from brain RNA-Seq data.

We found three genetic clusters using spleen data (**Figure 2A**): French Guiana (native range) formed its own genetic cluster, Hawaii (source) clustered with QLD (core), and NT (intermediate) clustered with WA (invasion front). In our brain dataset, population differentiation seemed to align with environmental barriers (**Figures 2B, C**): Hawaii (source) clustered with coastal QLD (core), whereas inland QLD/NT (intermediate) clustered with WA (invasion front). Inference of substructure within these two genetic clusters revealed that source and core toads may further differentiate into two separate groups (**Figure 2D**); however, intermediate and frontal toads remain one genetic cluster (**Figure 2E**). Removing candidates for selection (F_ST_ outliers and environmentally associated loci) from the analysis produced identical results (data not shown).

**Figure 3:**
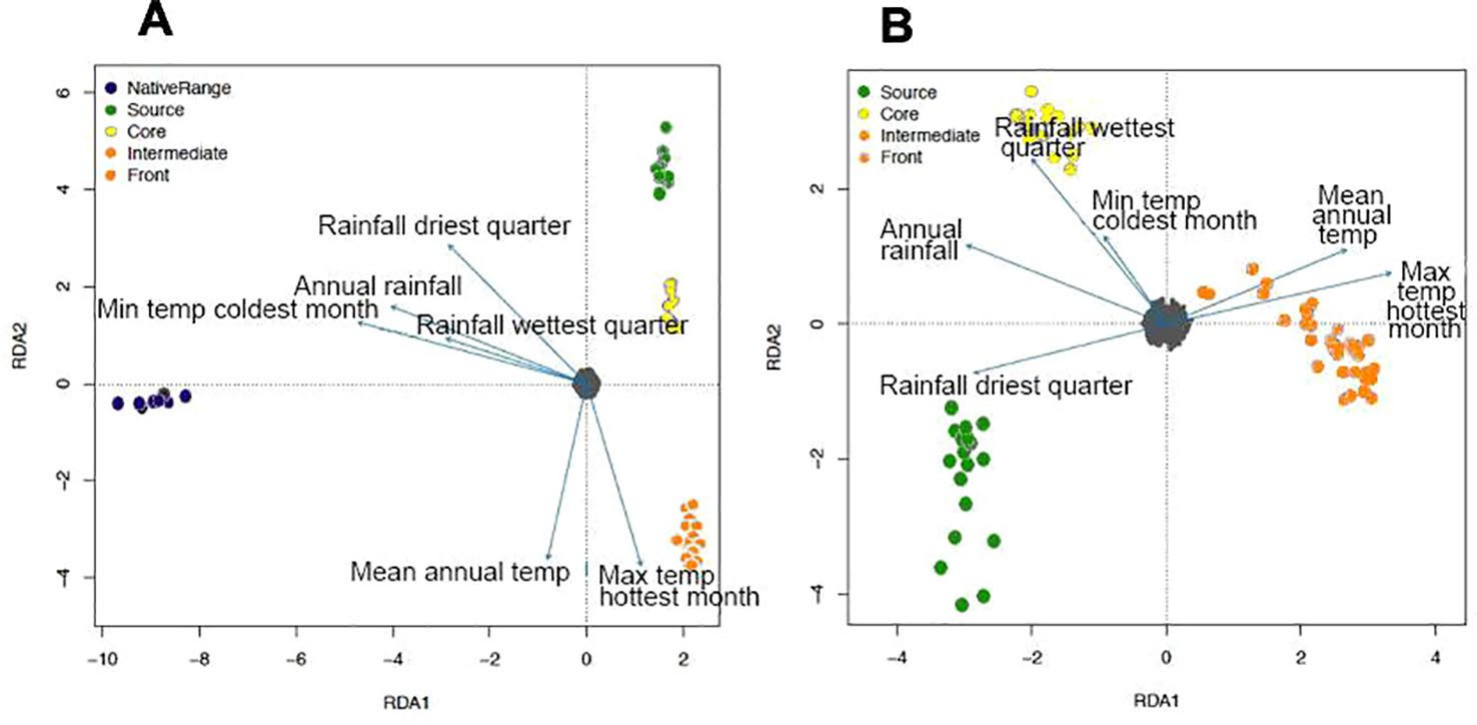
**(A)** Relationship between environmental variables and population structure of cane toads from French Guiana, Hawaii, and Australia, as inferred by redundancy analysis (RDA) on single nucleotide polymorphisms (SNPs) from spleen RNA sequencing (RNA-Seq) data. Environmental data include: annual mean temperature, maximum temperature of the warmest month, minimum temperature of the coldest month, annual precipitation, precipitation of the wettest quarter, and precipitation of the driest quarter. Small gray circles represent SNP loci. **(B)** Relationship between environmental variables and population structure of invasive cane toads from Hawaii and Australia, as inferred by redundancy analysis on SNPs from brain RNA-Seq data.

RDA was mostly consistent with fastStructure for both spleen (**Figure 3A**) and brain (**Figure 3B**) data, except for the differentiation of source toads and core toads as two separate genetic clusters. With the inclusion of the genetically distant native-range population in RDA on spleen data, most correlations between genetic structure and environmental variables were weak. However, divergence of intermediate and frontal toads was correlated with maximum temperature during the hottest month and mean annual temperature. Additionally, divergence of source toads appears to be related to rainfall during the driest quarter. RDA on brain data from only Hawaiian and Australian toads clarify this situation, suggesting that: 1) divergence of source toads from toads of other clusters is correlated with rainfall during the driest quarter. 2) Divergence of core toads from toads of other clusters is correlated with rainfall during the wettest quarter, mean annual rainfall, and minimum temperature during the coolest month. 3) Divergence of intermediate and frontal toads from toads of other clusters is correlated with maximum temperature during the hottest month and mean annual temperature.

### Tests for Selection

Out of 65,195 SNPs from spleen data, 648 were identified as F_ST_ outliers, with a mean global F_ST_ of 0.89. Mean pairwise F_ST_ values across outlier loci were 0.93 between the native and source/core populations, 0.97 between the native and intermediate/front populations, and 0.34 between the source/core and intermediate/front populations. Also from the spleen dataset, 3,088 SNPs were associated with maximum temperature during the warmest month and 1,813 were associated with rainfall during the driest quarter. Of these loci, 772 were associated with both environmental variables. We focused on SNPs that were detected both as F_ST_ outliers and environmental correlates: there were 64 outlier SNPs associated with maximum temperature and 47 outlier SNPs associated with rainfall in the driest quarter (including 18 outliers associated with both environmental variables).

Most of the 64 outlier SNPs associated with maximum temperature during the hottest month (**Table S3A**) were in transcripts with functions such as metabolism, transcription regulation, and immune function. There were also two SNPs in *HSP4*, a gene encoding heat shock protein (HSP) 4 (70 kDa), which is involved in thermal tolerance (on exposure to heat stress) and protein folding and unfolding (Consortium, 2017). Most of the 47 outlier SNPs associated with rainfall during the driest quarter (**Table S4A**) were in transcripts with functions such as cell signaling and immune function.

Out of 35,842 SNPs from brain data, 203 were identified as F_ST_ outliers, with a mean global F_ST_ of 0.37. Mean pairwise F_ST_ was 0.50 between the source/core and intermediate/front populations. Also from the brain dataset, 345 SNPs were associated with maximum temperature during the warmest month and 194 were associated with rainfall during the driest quarter. Nine of these loci were associated with both environmental variables. Of SNPs that were detected both as F_ST_ outliers and environmental correlates, there were 38 outlier SNPs associated with maximum temperature and 33 outlier SNPs associated with rainfall in the driest quarter; no outliers were associated with both environmental variables.

Most of the 38 outlier SNPs associated with maximum temperature during the hottest month (**Table S3B**) were in genes involved in cell signaling, mitochondrial processes (metabolism), or gene expression (Consortium, 2017). Similarly to the spleen data, there was one SNP from brain data in *HSP4*. Another SNP was in *ARNT2*, a gene encoding aryl hydrocarbon receptor nuclear translocator 2, a transcription factor involved in the hypothalamic pituitary adrenal (HPA) axis and visual and renal function (Consortium, 2017). Of the 33 outlier SNPs from brain data correlated with rainfall during the driest quarter (**Table S4B**), 19 were in *MAGED2*, a gene encoding melanoma-associated antigen D2, which is involved in renal sodium ion absorption (Consortium, 2017). Four were in *STXBP1*, a gene encoding syntaxin-binding protein 1, which is involved in platelet aggregation and vesicle fusion (Consortium, 2017). The rest were in genes generally involved in gene expression and cell signaling (Consortium, 2017).

### Evaluation of Genetic Differentiation, Diversity, and Isolation by Distance

Our spleen data show strong divergence between the native and source/core populations (pairwise F_ST_ = 0.29) based on the full SNP dataset, and even stronger divergences between native and intermediate/frontal populations (pairwise F_ST_ = 0.32). Differentiation between the source/core and intermediate/frontal populations was much lower (pairwise F_ST_ = 0.08). Overall divergence among natives and invaders was moderately high (global F_ST_ = 0.18).

In brain data, genetic differentiation between the source/core and intermediate/frontal populations also was low (pairwise F_ST_ = 0.07) based on the full SNP dataset. Differentiation between source and core toads was lower when calculated with brain data as compared to spleen (pairwise F_ST_ = 0.04). Generally, pairwise F_ST_ by invasion phase (**Table S5A**) and collection site (**Table S5B**) calculated from brain data revealed that toads from areas that are more closely linked by invasion history are less strongly differentiated. Because the brain data did not include native range samples, overall divergence was estimated to be much lower than it was in the spleen data (global F_ST_ = 0.04). In spleen and brain data, the distribution of FST values across the full dataset is wider than the distribution of FST values from the outlier dataset (**Figure S2**).

Diversity statistics from spleen data suggest that the native range population is only slightly more genetically diverse than the source/core population (native: He = 0.27, AR = 1.74, SI = 0.40; source/core: He = 0.26, AR = 1.74, SI = 0.40; *p* = 5E−4 for He) but both are more diverse than the intermediate/frontal population (He = 0.23, AR = 1.68, SI = 0.36; *p* < 2E−16 for all measures). However, we also estimated these measures by region (**Table 1**) or collection site (**Table S6**) rather than by genetic cluster to detect changes in genetic diversity at a finer scale; these calculations reveal a more obvious decline in genetic diversity from the native population to all invasive collection sites in all loci analyzed.

Despite this reduction in genome-wide genetic diversity, subsets of loci putatively under selection in the spleen dataset showed no loss of diversity during introduction (**Table 1**). Here there was an increase in diversity in the source/core population as compared to native samples (native: He = 0.03, AR = 1.25, SI = 0.06; source/core: He = 0.09, AR = 1.37, SI = 0.14; *p* < 2E−16 for all measures). At these same loci, diversity was lowest in the intermediate/front population (He = 0.02, AR = 1.14, SI = 0.04). These trends are also shown in environmentally associated loci (native: He = 0.17, AR = 1.53, SI = 0.25; source/core: He = 0.37, AR = 1.99, SI = 0.55; intermediate/front: He = 0.23, AR = 1.81, SI = 0.35). When calculated by region (**Table 1**) or collection site (**Table S6**) rather than by genetic cluster, the trends remain the same.

Genome-wide diversity estimates using brain data show similar patterns to spleen data (source/core: He = 0.34, AR = 2.00, SI = 0.51; intermediate/front: He = 0.31, AR = 1.97, SI = 0.47; *p* < 2E−16 for comparisons of all measures; **Table 2**). This reduction is greater in loci with outlier F_ST_ values (source/core: He = 0.38, AR = 2.00, SI = 0.55; intermediate/front: He = 0.15, AR = 1.70, SI = 0.24; *p* < 2E−16 for comparisons of all measures) and in environmentally associated loci (source/core: He = 0.35, AR = 2.00, SI = 0.53; intermediate/front: He = 0.08, AR = 1.57, SI = 0.15; *p* < 2E−16 for comparisons of all measures). When calculated by region (**Table 2**) or collection site (**Table S7**) rather than by genetic cluster, the trends remain the same.

**Figure 4:**
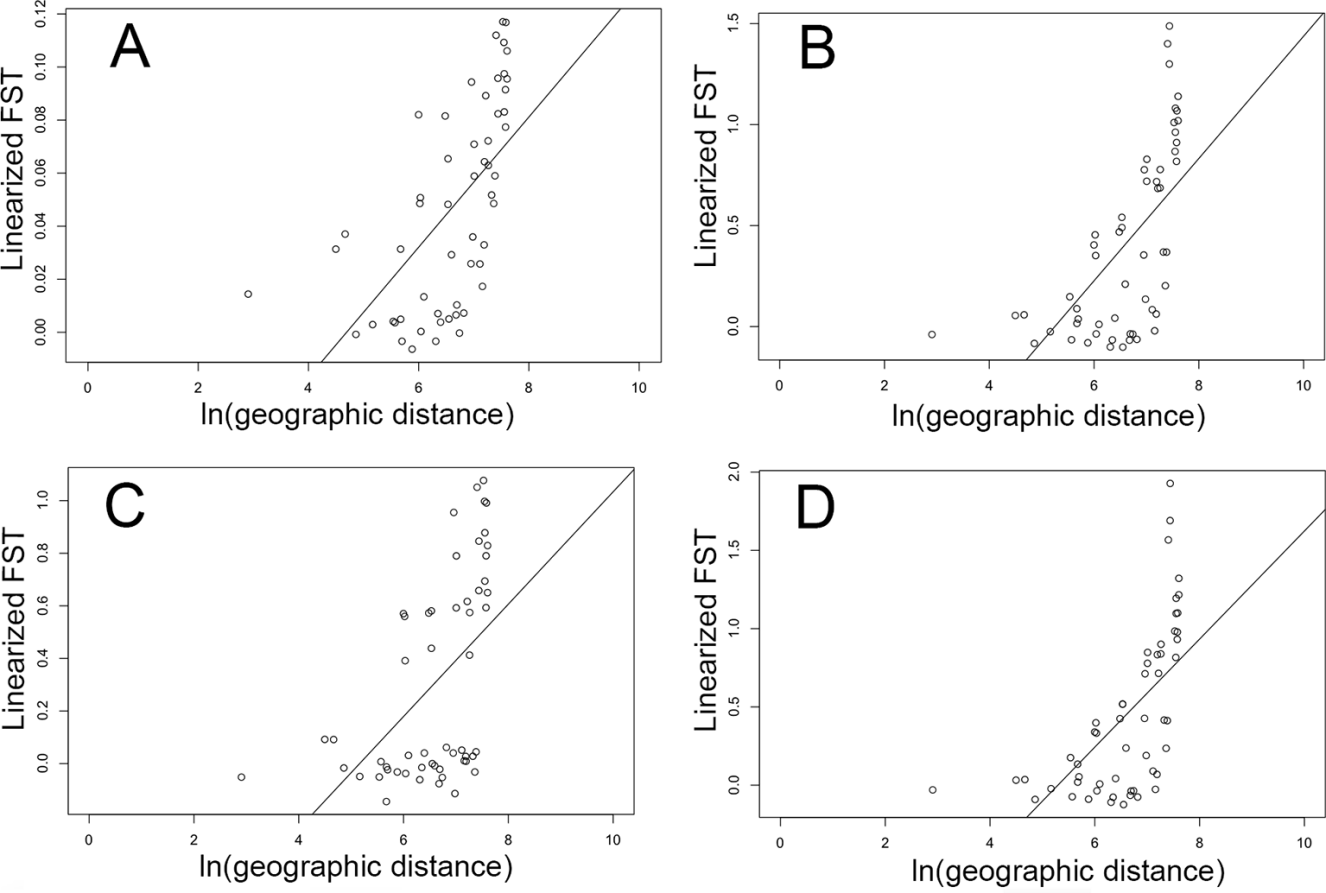
Relationship between geographic distance (natural log of km) and genetic distance (linearized pairwise F_ST_) between cane toads collected across their invasive Australian range (**Figure 1**) as inferred by brain RNA-Seq data. **(A)** represents the full dataset of loci (*p* = 1E−3, *r* = 0.61). **(B)** represents all F_ST_ outliers (*p* = 1E−3, *r* = 0.63). **(C)** represents only F_ST_ outliers with an association with either rainfall or temperature (*p* = 1E−3, *r* = 0.52). **(D)** represents only F_ST_ outliers without an association with either rainfall or temperature (*p* = 1E−3, *r* = 0.63).

Geographic distance in collection sites and pairwise F_ST_ values by collection site (as estimated with brain data) were significantly associated across all four of our Mantel tests using: 1) all loci (*p* = 1E−3, *r* = 0.61; **Figure 4A**); 2) all loci with outlier F_ST_ values (*p* = 1E−3, *r* = 0.63; **Figure 4B**); 3) only loci with outlier F_ST_ values and an environmental association (*p* = 1E−3, *r* = 0.52; **Figure 4C**); 4) only loci with outlier F_ST_ values but without an environmental association (*p* = 1E−3, *r* = 0.63; **Figure 4D**).

### Single Nucleotide Polymorphism Annotations

Annotation of SNPs revealed that most of our loci were either missense, synonymous, or found in the 3' untranslated region (UTR). A small percentage were found in the 5' UTR, and the remaining loci involved the loss or gain of stop or start codons (**Figures S3** and **S4**). In the spleen data, the temperature-associated outlier F_ST_ subset had a significantly higher proportion of synonymous variants (*p* = 8E−3) than the full set of SNPs. The outlier loci within *HSP4* were both synonymous variants. However, there were no significant differences in proportions between the rainfall-associated outlier F_ST_ subset and the full set of SNPs.

In the brain data, there were no significant differences in proportions of SNP variant types between the temperature-associated outlier F_ST_ subset and the full set. The locus within *HSP4* was a 3' UTR variant, and the locus within *ARNT2* was synonymous. The rainfall-associated outlier F_ST_ subset had a significantly higher proportion of 3' UTR variants (*p* = 5E−3) and lower proportion of missense variants (*p* = 0.04) than did the full set. Eighteen of the nineteen loci within *MAGED2* were 3' UTR variants (the last was missense), and two of the four loci within *STXBP1* were missense (the other two were synonymous).

### Population Structure in Australian Toads From the Restriction Site-Associated Deoxyribonucleic Acid Sequencing Experiment

**Figure 5:**
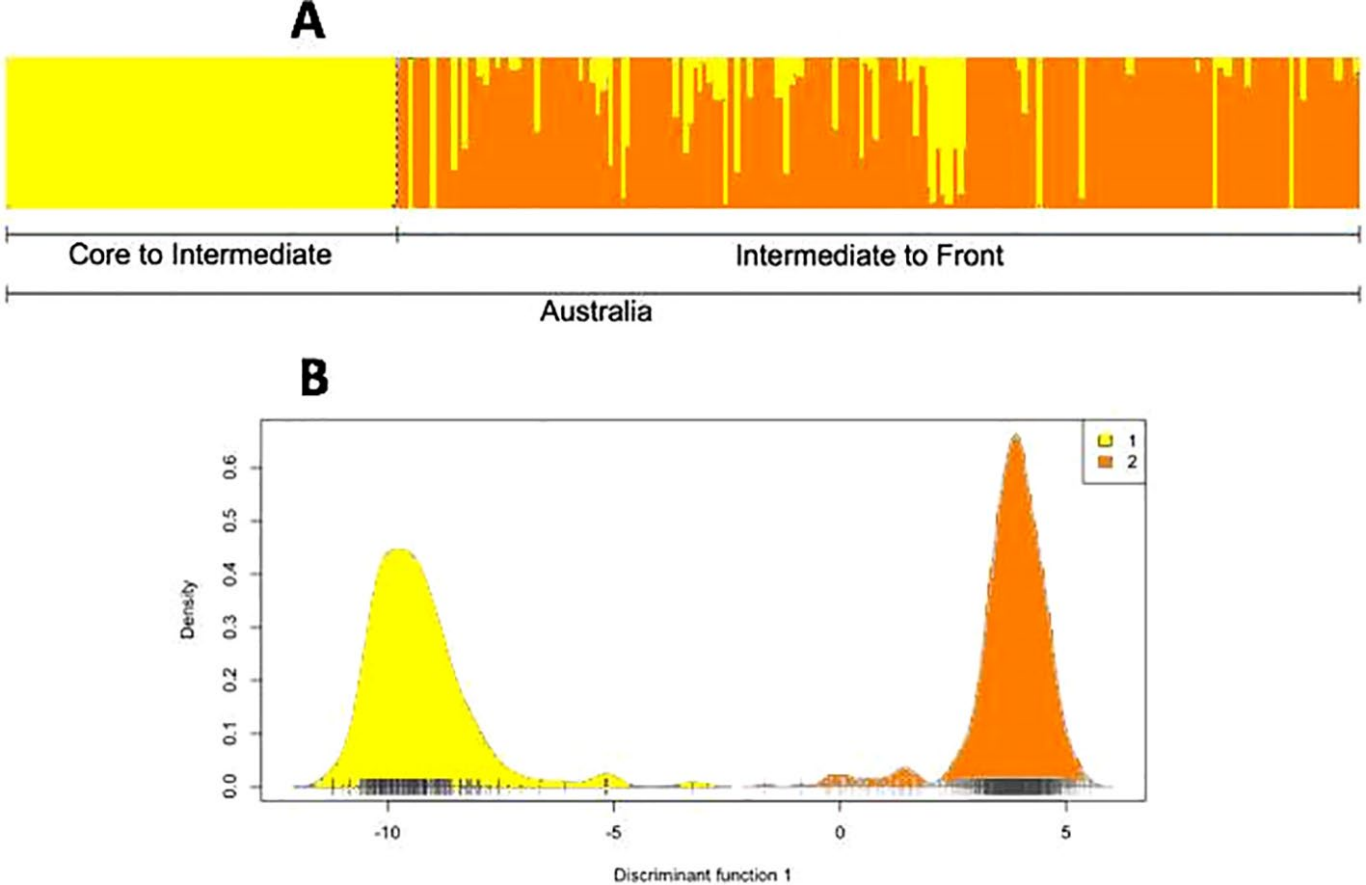
**(A)** Population structure of cane toads across their invasive range in Australia, as inferred from running fastStructure on single nucleotide polymorphisms (SNPs) from restriction site-associated DNA sequencing (RADSeq) data. Because coordinate metadata for collection sites were unavailable, the samples could not be arranged from east to west along the Australian range. **(B)** Population structure of cane toads across their invasive range in Australia, as inferred from discriminant analysis of principal components on SNPs from RADSeq data.

Analysis of the RADSeq dataset through fastStructure and DAPC also showed two genetic clusters within Australia (**Figures 5A, B**): QLD (core toward intermediate areas) in the first group, and the border of NT/WA (intermediate areas toward the invasion front) in the second group. There was little differentiation between these two groups (pairwise F_ST_ = 0.09) or overall (global F_ST_ = 0.04). We found no evidence of significant substructure within either of the two genetic clusters. The two groups were equal in allelic richness (AR = 2.00 for both groups), but expected heterozygosity was higher in core toads than in intermediate/frontal toads (core: He = 0.50; intermediate/front: He = 0.33).

## Discussion

In this study, we tested whether cane toads fit the commonly invoked “genetic paradox of invasion, ” leading to our prediction of lower genetic diversity in invasive populations compared to those in the native range. Our analyses show evidence of a reduction in genetic diversity from native to invasive populations across all loci. We also predicted strong divergence between native range and invasive populations resulting from genetic drift, selection, and spatial sorting. We found three genetic clusters: 1) native range toads, 2) Hawaiian source toads and eastern Australian range core toads, and 3) all toads from further west in more recently colonized areas (intermediate and invasion front). Differentiation is much higher between the native population and the invasive populations than between the invasive populations. Loci identified as F_ST_ outliers had values greater than 0.9 between native and invasive populations, indicating that many of these loci are likely to be fixed for different alleles in these populations. Within Australia, eastern and western genetic groups separated by the continental divide are consistent with selection in the more arid environment inhabited by western toads. However, the presence of isolation by distance across all loci also supports gradual genetic drift across invasion phases; that this relationship is strengthened in the subset of F_ST_ outliers without environmental associations may indicate the presence of spatial sorting. It seems likely that all these evolutionary forces have shaped Australian invasive populations.

Despite these findings, evidence that invasive cane toads fit the genetic paradox paradigm is equivocal. We did identify a reduction in overall genetic diversity, both in allelic richness and evenness (He and SI). Previous studies on mitochondrial DNA (Slade and Moritz, 1998) and microsatellite (Estoup et al., 2001) data have also suggested losses in genetic diversity from the native range to Hawaii and Australia. However, we did not find this reduction in the subset of loci putatively under selection (loci with outlier F_ST_ values or associations with environmental variables through LFMM; **Table 1**). This supports the prediction that some invasions do not represent a true genetic paradox; despite overall loss of genetic diversity and the presence of novel adaptive challenges, genetic diversity at ecologically relevant traits may be maintained by balancing selection (Estoup et al., 2016). Diversity of this subset of loci is not only maintained in the source/core population; it is higher than in the native population. This may be due to the small sample size (N = 8) of the native-range population in this study; but this pattern remains when separating the source and core populations, and their sample sizes are only slightly higher (N = 10 each). Alternatively, this may reflect admixture from multiple genetically distinct introductions; the Hawaiian introduction was sourced from the Puerto Rican population, which included introductions from French Guiana and Guyana (Turvey, 2009). Finally, it is possible that *de novo* mutation rates may have been high enough to restore or even enhance adaptive potential in the source/core population (Estoup et al., 2016). From the source/core population to the intermediate frontal population, however, diversity of loci putatively under selection is highly reduced; we believe this is because of directional selection, as outlined below.

Our two invasive genetic clusters (source/core and intermediate/front) diverge at the transition from coastal to inland areas within Australia, in an area where temperatures become higher and rainfall becomes scarcer, particularly during the dry season (Bureau of Meteorology, A.G, 2018). The correlation between population structure and climatic patterns supports the hypothesis of adaptation, which is further reinforced by RDA results. Heterogeneous climates across introduced or expanding ranges have previously been linked to genetic differentiation, possibly due to climatically imposed selection (Weber and Schmid, 1998; Leydet et al., 2018).

Diversity estimates from our brain data suggest a reduction in diversity across the invasion in the full set of loci, but a much greater reduction in diversity in those identified as under selection (**Table 2**). The source (Hawaii) and core (QLD) areas represent earlier phases of the invasion and experience similar environmental conditions to those of the native range, whereas intermediate (NT) and invasion front (WA) areas are more arid. Thus, the greater depletion in diversity at outlier loci across the Australian invasion may be due to the effects of directional selection from the harsher climate at more recently colonized areas of the Australian range (Andrews, 2010a; Estoup et al., 2016).

Among our F_ST_ outliers associated with temperature are several within a gene encoding protein HSP 4 (70 kDa). HSPs protect protein folding during increased temperatures and provide cells with enhanced thermal tolerance (Kiang and Tsokos, 1998), and expression of HSP genes has been shown to underlie adaptive responses to environmental stress (Chen et al., 2018). Changes in HSP levels in response to thermal environment manipulation vary between native and invasive cane toads, as well as between populations of toads within Australia (Kosmala et al., 2018b). In our study, the SNPs within *HSP4* included two synonymous variants (spleen data) and a 3' UTR variant (brain data); 3' UTR variants are known to alter levels of protein functioning through differential expression of genes (Skeeles et al., 2013), and synonymous mutations have also been shown to increase fitness through increased gene expression (Bailey et al., 2014). In the case of HSPs, adaptation may not necessarily mean a change in function, but rather a change in the expression levels of proteins involved in the function, which could be caused by a 3' UTR variant or a synonymous mutation.

Aryl hydrocarbon receptor nuclear translocator 2 (from the *ARNT2* gene, also associated with temperature), is a transcription factor that specifically acts on genes involved in the HPA axis and visual and renal function. The HPA axis is stimulated by high temperatures (Malmkvist et al., 2009), and the secretion of corticosterone can be modulated adaptively in response to thermal change (Telemeco and Addis, 2014). Elevation of corticosterone secretion in toads increases the rate of desiccation, thus making it maladaptive in arid environments (Jessop et al., 2013). Corticosterone levels in Brazilian and Australian toads in response to manipulation of thermal environment display similar patterns to those of HSPs (Kosmala et al., 2018b). Selection on loci potentially affecting corticosterone secretion by affecting the HPA is consistent with these results, and the SNP we investigated in *ARNT2* is synonymous, which may influence HPA activation by causing changes in expression.

More than half (19 of 33) of the F_ST_ outliers associated with rainfall (brain data) were found in *MAGED2* (encodes melanoma-associated antigen D2). This protein regulates the expression and localization of two co-transporters that facilitate renal sodium ion absorption, preventing excess loss of water and solutes *via* reabsorption through the kidneys (Greger, 2000). Similarly to the SNP within *HSP4*, 18 of 19 SNPs within *MAGED2* were 3' UTR variants, which can affect gene expression (Skeeles et al., 2013). The high number of SNPs found in the 3' UTR of *MAGED2* is likely responsible for the difference in proportions of variant types between the full dataset and the rainfall-associated outlier dataset. Four SNPs (including missense variants) were found in *STXBP1* (encodes syntaxin-binding protein 1, which is involved in vesicle fusion and blood clotting). Dehydration is known to increase blood clotting rate (El-Sabban et al., 1996), and excessive blood clotting can be harmful to the heart, brain, and limbs (PubmedHealth, 2014). Evolved changes in the rates of renal sodium absorption and blood clotting may allow toads from intermediate and invasion front areas to survive in drier environments. The remaining SNPs associated with rainfall or temperature were in genes generally involved in gene expression or signal transduction; these genes may facilitate expression of (or signaling to) the genes we have discussed, or may have independent functions in cellular maintenance.

Invasive species have been shown to rapidly adapt phenotypically to heterogeneous climatic conditions, enabling range expansion (Colautti and Barrett, 2013). Some invaders tolerate higher temperatures (Braby and Somero, 2006) and water loss (Godoy et al., 2011) better than do related indigenous taxa (Zerebecki and Sorte, 2011). There is evidence of this in cane toads: under dehydrating conditions, wild-caught individuals from Australia exhibit better locomotor performance than do conspecifics from Hawaii and the native range (Kosmala et al., 2017), and within Australia, toads from semi-arid areas (i.e., NT) exhibit better locomotor performance than do conspecifics from wetter areas (i.e., coastal QLD) (Tingley et al., 2012; Kosmala et al., 2017). This trait is heritable; captive-bred toads with parental origins from hotter areas (northwestern Australia) outperform those with parental origins from cooler areas (northeastern Australia) at high (but not low) temperatures in a common-garden setting (Kosmala et al., 2018a). Coupled with the previously shown heritable phenotypic patterns, our results suggest that intermediate and frontal toads in Australia may be evolving enhanced thermal tolerance, thereby facilitating their continued westward range expansion (Szucs et al., 2017). However, manipulative experiments are required to directly test this hypothesis.

We found evidence of isolation by distance (IBD) in Australia using the full SNP dataset, which is likely to be caused by genetic drift. However, loci under strong selection are less likely to show evidence of IBD. Unsurprisingly, when we plot genetic *vs.* geographic distance in Australia, using loci putatively associated with environmental selection, we see a clear discontinuity in these data (**Figure 4C**). Other evolutionary forces may be more difficult to detect in these data. Spatial sorting is expected to result in clinal variation in dispersal-related allele frequencies along the range, so we might expect that the relationship between genetic and geographic distance using loci underlying dispersal-related characteristics would show a similar pattern to that of genetic drift. In this study, when we examine this relationship only using loci identified as under selection, but not associated with environmental selection, there is a gradual decline in the relationship between genetic and geographic distance (**Figure 4D**). Although these loci may also be influenced by drift to some degree, it seems plausible that because they were identified as F outliers, they may be influenced by other evolutionary forces, including spatial sorting. Additionally, demographic events may have played a role in shaping genetic variation in this system.

Our population structure analyses using the RADSeq dataset (Trumbo et al., 2016) yielded results similar to those from our RNA-Seq dataset (two genetic clusters within Australia). However, unlike the RNA-Seq results (which show a divide occurring in inland QLD, where temperature increases and rainfall decreases), the RADSeq results cluster all QLD toads in one group, and NT/WA toads in another. These differences between the two datasets may result from selection driven by environmental variables in the RNA-Seq data, whereas demographic processes may lessen differentiation between sites in the RADSeq data. Further, toads included in the RADSeq dataset were sampled farther south than those in our study, and the environmental conditions of coastal and inland sites are more similar in that area (Hijmans et al., 2005). Diversity estimates from the RADSeq dataset were slightly higher than those from the RNA-Seq dataset, which may have occurred because adaptive variation is expected to be lower than neutral variation due to evolutionary constraints (Andrews, 2010a).

In conclusion, it appears that serial introductions have led to a reduction in genetic diversity of invasive cane toads. Nonetheless, cane toads do indeed face novel adaptive challenges in their introduced ranges, and the loci that we have identified as putatively under selection may suggest that they are able to respond genetically to natural selection, particularly in the harsh environmental conditions of northwestern Australia. The ability of toads to adapt to these conditions may reflect the maintenance of diversity at ecologically relevant loci, or it may reflect sufficiently high mutation rates to bolster adaptive potential; thus, it remains unclear whether the toad invasion represents a true genetic paradox. It seems likely that natural selection, genetic drift, and spatial sorting all shape the Australian invasive population, but current approaches do not have the power to distinguish between these processes due to their potentially similar effects on allele frequencies. Manipulative experiments investigating the candidates that we have identified may be especially informative. Additionally, genetic changes may not be the only mechanisms by which adaptation occurs; studies on plasticity and epigenetic changes may also be useful for uncovering the mysteries of rapid evolution.

## Data Availability Statement

The datasets generated for this study can be found in Sequence Read Archive (SRA), BioProject Accession PRJNA510261 (spleen data from French Guiana and Hawai’i), PRJNA395127 (spleen data from Australia), and PRJNA479937 (all brain data).

## Ethics Statement

All procedures involving live animals were approved by the University of Sydney Animal Care and Ethics Committee (2014/562) and the Deakin University Animal Ethics Committee (AEX04-2014).

## Author Contributions

DS designed the experiment, performed the lab work and bioinformatics analyses, and wrote the paper. MR collected samples, assisted with the experimental design and bioinformatics analyses, and edited the paper. RS assisted with the experimental design and edited the paper. JD and SD collected samples and edited the paper. LR collected samples, assisted with the experimental design and bioinformatics analyses, and edited the paper.

## Funding

This work was supported by the Australian Research Council (FL120100074, DE150101393) and the Equity Trustees Charitable Foundation (Holsworth Wildlife Research Endowment).

## Conflict of Interest

The authors declare that the research was conducted in the absence of any commercial or financial relationships that could be construed as a potential conflict of interest.
